# Religious change preceded economic change in the 20th century

**DOI:** 10.1126/sciadv.aar8680

**Published:** 2018-07-18

**Authors:** Damian J. Ruck, R. Alexander Bentley, Daniel J. Lawson

**Affiliations:** 1 Population Health Sciences, University of Bristol, Oakfield House, Bristol BS8 2BN, UK.; 2 Anthropology Department, University of Tennessee, 1621 Cumberland Avenue, Knoxville, TN 37996, USA.

## Abstract

The decline in the everyday importance of religion with economic development is a well-known correlation, but which phenomenon comes first? Using unsupervised factor analysis and a birth cohort approach to create a retrospective time series, we present 100-year time series of secularization in different nations, derived from recent global values surveys, which we compare by decade to historical gross domestic product figures in those nations. We find evidence that a rise in secularization generally has preceded economic growth over the past century. Our multilevel, time-lagged regressions also indicate that tolerance for individual rights predicted 20th century economic growth even better than secularization. These findings hold when we control for education and shared cultural heritage.

## Teaser

Religious change predicted economic change in the 20th century, across the world.

## INTRODUCTION

A classic sociological question is whether the decline of religious activity, or secularization, has been caused by economic development (*1*, *2*). A century ago, Durkheim (*3*) proposed that technological and socioeconomic advances come to displace the functions of religion (*4*, *5*), whereas Weber (*6*) contended the opposite, that monotheistic religion—the so-called Protestant ethic—made the development of capitalism possible.

Although a correlation between economic development and secularization is evident, in that countries that are highly religious tend to be the poorest (*7*, *8*), it is not obvious which change precedes which through time: whether development causes secularization (*9*, *10*), or vice versa (*11*), or whether both changes are driven, with different time lags, by a factor such as education or advances in technology (*2*, *12*).

Whereas some studies find a bicausal relationship between income and religion (*13*–*15*), causality effectively remains unknown as feedbacks may change through time and development. Organized religious charity, for example, might initially encourage certain values that facilitate economic development while restricting individual expression (*16*), but then the resulting economic development may subsequently reward individualism. Tolerance of individual expression may then feed secularism, partly by undermining religious organizations that provide communities with resources and social capital; other causal arguments also remain feasible (*17*, *18*).

To characterize their temporal relationship, we use 20th century data for both economic development and a measure of secularization extracted from international cultural values surveys. Time-lagged regressions using these 100-year time series for key variables can determine which variable precedes the other. This can be used to rule out certain hypotheses of causality. If, for example, changes in series *X* precede those in series *Y*, then one can say that *Y* does not cause *X* even while it is not certain that *X* causes *Y*.

For indices of cultural values, we use data from the European Values Survey (EVS) and the World Values Survey (WVS) since 1990. To estimate values from all decades of the 20th century, here, we make use of birth cohorts (see Materials and Methods); the utility of this technique is among our key findings. The WVS and EVS featured some of the same survey questions, so we combined these survey data sets, which we refer to as the WEVS. The data we use for economic development are data on historic gross domestic product (GDP) per capita from the Maddison Project (*19*), which, although more complex indices such as the Human Development Index can be preferable (*20*), we use because GDP data exist for many nations over the entire 20th century. We also include three control variables, each of which covers the set of nations around the world and extends to the beginning of the 20th century. The first, which recently became available, is an extensive international time series on participation rates in tertiary education, which stretches back to the early 20th century (*21*). The second is language family, which is used as a proxy for the nested random effect of nonindependent cultural and economic histories between individual countries (*22*). The third is a measure of tolerance of others, extracted as a factor from the WEVS, as discussed below.

## RESULTS

Having applied exploratory factor analysis (EFA) to the WEVS data set, we selected nine factors to retain, each interpretable using questions that did not overlap between factors (see Materials and Methods). Factor 1, explaining 11% of the variance, was strongly loaded on questions regarding the importance of religion in one’s life (see Materials and Methods). This factor defines our measure of secularization, *S*, as a composite variable of WEVS responses (table S4 lists all elements of the secularization factor).

Another factor from the EFA, factor 8, was strongly loaded on survey participants’ willingness to tolerate behaviors that are often socially prohibited, such as suicide, homosexuality, or abortion (see table S11). We label this factor as “tolerance,” denoted as *V*. We explore tolerance, *V*, as a control variable in our results for two reasons. First, as we will see, the changes in tolerance were closely correlated with the changes in secularism during the 20th century. Second, the tolerance factor was strongly correlated (*R* = 0.59) with Hofstede’s (*23*) metric of individualism (we did not extract individualism directly from Hofstede’s data because it is not broken down by birth cohort as we require).

Having extracted these factors measuring secularization, *S*, and tolerance, *V*, we aim to extend the information collected in the WEVS during the last quarter-century back to the beginning of the 20th century. To do this, we treat decade of birth, *t*, recorded for each person surveyed, as a proxy for a historical time period. Although there will be differences from one survey period, *p*, to the next, the differences apply across all birth cohorts such that the relative differences between birth cohorts were generally maintained through time (*5*).

Using a likelihood ratio test of the hypotheses presented in Materials and Methods, we confirm that estimates for each birth decade, *t*, are independent of survey period, *p*, in that there is no evidence of temporal dependence for *S*_*t*,*p*_ or for *V*_*t*,*p*_ in 91% and 89% of the countries tested, respectively (table S13 shows full results). This confirms that generational trends persist through time. The results make up the elements of an array, *S*_*i*,*t*,*p*_, of estimates of secularization in each country, *i*, during birth decade, *t*, for survey period, *p*.

**Fig. 1:**
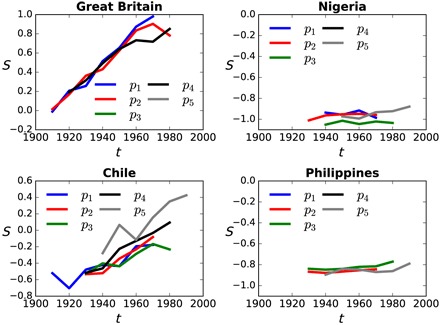
Temporal trends in secularization versus economic development over the 20th century, for four illustrative countries. Each panel represents a country’s secularization score *S* derived from the WEVS on the *y* axis, for birth cohorts by decade *t* on the *x* axis. The trends are independently determined from each of five different survey periods, *p*, corresponding to five waves of the WEVS: *p*_1_, 1990–1994; *p*_2_, 1995–1999; *p*_3_, 2000–2004; *p*_4_, 2005–2009; *p*_5_, 2010–2014.

Next, we determined secularization by birth decade in each country, *S*_*i*,*t*_, by averaging factor 1 across all available survey periods, *p*, in country, *i*, corresponding to decade of birth, *t*. Figure 1 illustrates the temporal trend in secularization across birth decade for several countries with little missing data: Great Britain, the Philippines, Chile, and Nigeria. For countries with missing data, missing values of *S*_*i*,*t*_ were imputed (see Materials and Methods). The same procedure is used to obtain the tolerance score matrix *V*_*i*,*t*_.

**Fig. 2:**
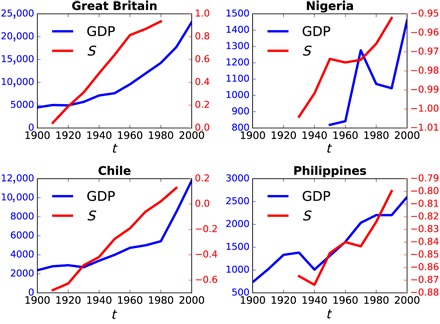
Time series of secularization versus GDP per capita, from four illustrative countries, over the 20th century. Each red line represents the mean secularization score, *S*_*t*_, of the birth cohort in decade, *t*, for that country. Each blue line represents the mean GDP per capita (normalized to 1990 US$) during decade *t* in that country.

We compared *S*_*i*,*t*_ versus historical GDP per capita (in 1990 US$) from each country through time. Figure 2 compares *S*_*i*,*t*_ versus the decadal mean GDP, GDP_*i*,*t*_, for the same four countries as Fig. 1. We find evidence that changes in secularization, *S*_*i*,*t*_, precede changes in GDP_*i*,*t*_, as most clearly seen in reversals of the trend, when a decrease in *S* occurs shortly before a corresponding decrease in GDP.

To test whether changes in *S*_*i*,*t*_ generally precede changes in GDP_*i*,*t*_, or vice versa, we estimate multilevel time-lagged regressions. By including data from all countries in a single test, a multilevel model can maximize the statistical power available in these data. It also allows us to control for non-independence due to shared cultural heritage *h*, which we do by classifying countries by language family (see Materials and Methods). The multilevel model is

$$S_{t}\sim S_{t-y}+{\text{GDP}}_{t-y}+(1|h_{i})+\epsilon$$ (1)

$${\text{GDP}}_{t}\sim {\text{GDP}}_{t-y}+S_{t-y}+(1|h_{i})+\epsilon$$ (2)

where *S*_*t*_ and GDP_*t*_ are secularization and economic development in decade *t*, respectively, and *S*_*t*−*y*_ and GDP_*t*−*y*_ are the respective values lagged by *y* decades. The term *h*_*i*_ represents a nested random effect due to the cultural-historic grouping of country *i*, for which language family is the proxy (*22*). This term is used as a control for non-independent similarities between individual countries, due to their development and secularization already present at the start of the 20th century.

Models 2 and 5 (Table 1) show that changes in *S*_*t*_ precede those of GDP_*t*_ and not the other way around. This directionality is independent of time lag, *y*, as the full results for lags of one decade, two decades, and three decades show (table S13). An increase in *S*_*t*_ by 1 SD corresponds to $1000 increase in GDP_*t*_ per capita after 10 years, $2800 after 20 years, and $5000 after 30 years. Our robustness checks show that this result is stable (see Materials and Methods). It is independent of the age we consider a birth cohort economically active (table S15).

**Table 1:** Selected time-lagged linear regressions (labeled models M2, M5, etc.) between secularization (*S*), development (GDP), tolerance (*V*), and education (*E*). The time lag is *y* = 2 decades in all cases (results for *y* = 1, 2, and 3 decades in table S14). SEs, in parentheses, were determined from the inverse of the negative Hessian matrix (*44*). *N* is the number of data points for each autoregression, *n* is the number of countries included in the data set, *i* is the percentage of residual variance explained by the random effect (country), and *h* is the percentage explained by cultural heritage. *R*^2^ is the total variance explained. Bonferroni-corrected significance: *P < 0.1, **P < 0.05, ****P < 0.01.

**Model**	**M2**	**M5**	**M8**	**M11**	**M14**	**M17**
**Variable**	***S***	**GDP**	***S***	**GDP**	***S***	**GDP**
**Fixed effect**						
GDP_*t*−2_	−0.02 (0.03)	0.87 (0.04)***	−0.01 (0.03)	0.78 (0.04)***	−0.04 (0.05)	0.83 (0.05)***
*S*_*t*−2_	0.97 (0.02)***	0.28 (0.03)****	0.99 (0.04)***	0.01 (0.04)	0.97 (0.02)***	0.22 (0.02)***
*V*_*t*−2_			−0.02 (0.03)	0.32 (0.04)***		
*E*_*t*−2_					0.14 (0.2)	0.97 (0.19)***
**Random effect**						
*i*	0.14***	0.21***	0.14***	0.19***	0.15***	0.16***
*h*	0.14***	0.12*	0.14***	0.1	0.12*	0.09
**Summary**						
*R*^2^	0.99	0.89	0.99	0.9	0.99	0.91
*N*	324	469	324	469	274	382
*n*	95	101	95	101	69	70

Next, we tested whether the tolerance factor, *V*, offers any explanatory value, by adding it as a control in Eqs. 1 and 2 (see Materials and Methods). Model 8 in Table 1 shows that *V* is not predictive of future *S*, but model 11 shows that *V* is a better explanation of future GDP than *S*. This result is independent of time lag, an increase of 1 SD in *V* results in a $900 increase in GDP_*t*_ per capita after 10 years, $3200 after 20 years, and $4400 after 30 years (see table S14).

**Fig. 3:**
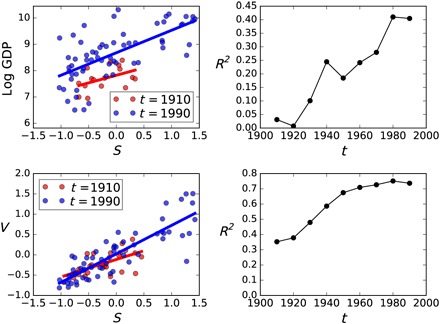
Emergence of the correlation between secularization and development during the 20th century. The top left panel shows scatter plots for secularization, *S*_*t*_, against log GDP_*t*_ per capita (normalized to 1990 US$), for people born in 1910 and 1990, where each point is a country. The top right panel shows *R*^2^ values for GDP_*t*_ versus *S*_*t*_, for the decades of the 20th century, *t*. The bottom left panel shows the same scatter-country plot for *S*_*t*_ against *V*_*t*_ for people born in the 1910s and the 1990s. The bottom left panel shows the progression of this correlation through the decades of the 20th century.

The top row of Fig. 3 compares the relationship between *S*_*t*_ and GDP_*t*_ in the 1910s and the 1990s, along with the evolution of this relationship during the 20th century. Whereas there was no relationship in the 1910s, a strong relationship had formulated by the 1990s; secularization explained only 4% of the variance in global development in the 1910s, but explained 40% in the 1990s. This contrasts with the 20th century relationship between *S*_*t*_ and *V*_*t*_ (Fig. 3, bottom row), in that they were already related in the 1910s—*S*_*t*_ explained 36% of the variance in *V*_*t*_ in the 1910s—which since increased to 72% by the 1990s. This suggests that the relationship between secularization and economic development did not exist during the 19th century and that the relationship between tolerance and secularization probably did.

Having ruled out economic development as a plausible cause for secularization, we test the effect of education, using a new international data set on enrollment in tertiary education since the 19th century (*21*). We added education as a control in Eqs. 1 and 2 (see Materials and Methods). The results show that higher education is a good predictor of future GDP but not of future secularization (Table 1, models 14 and 17). These results are robust to different time lags (see table S14).

To test noise effects in the EFA, we repeated the analysis by redefining *S*_*t*_ using an average of six subjectively identified variables. The results were the same (table S16).

## DISCUSSION

In this study, we have shown that, across a diversity of countries around the world, changes in secularization predicted changes in GDP worldwide during the 20th century. More broadly, this implies that changes in the everyday importance of religious practices preceded changes in economic development in the 20th century. While this does not yet isolate one path of causality, it determines that economic growth is not what caused secularization in the past. Our observation that secularization preceded economic change further rules out a bicausal relationship between income and religion (*13*–*15*) as well as the theory that socioeconomic advances cause religious practices to be phased out (*3*, *4*, *17*).

Our findings do not mean, however, that secularization was the ultimate cause of economic development. Both secularization and economic growth may have been driven by something else, with secularization responding faster than GDP. This likely rules out technological advances as the ultimate cause, as it is hard to imagine how religion could respond faster to technological change than GDP.

Tolerance of individual rights appears to be closer to an ultimate driver, in that more people are included in economic activity, especially women (*24*, *25*). The tolerance factor, which is most highly loaded on individual rights for divorce and abortion (table S11) and therefore likely to correlate with women’s rights generally, was a better temporal predictor of GDP per capita than the secularization factor. Although temporal changes in tolerance and in secularization were synchronous, secularization did not predict increased GDP in the absence of accompanying increases in tolerance. The tolerance factor also correlates with Hofstede’s individualism, which “has a strong and robust effect on log GDP per capita,” according to other studies (*26*).

Besides tolerance, education is a possible driver of both economic development and secularization. Our results showed that education is predictive of future GDP, but not of future secularization. This is consistent with other findings (*2*, *12*) and also with religious countries tending to have high support for science education (*17*). In nations where secular government programs gradually replace religious institutions as provider of education and social welfare, changes in education would tend to be subsequent (*27*, *28*).

Methodologically, our unsupervised approach to the WEVS data set, combined with the use of birth cohorts to extend the temporal reach of these data, is different from previous studies. Our analysis required new methods of unsupervised factor analysis and extraction of a century of temporal change from a much more recent data set. The evidence was derived by comparing, for different countries, historical GDP versus multifactor measures of personal values extracted and extrapolated from 25 years of the WEVS into a set of 100-year time series both for economic development and for a measure of secularization.

Previous studies have mainly focused on how education or personal income correlates with measures of religiosity such as church attendance in Western and/or European countries (*9*, *12*–*14*, *28*, *29*). Rather than choosing WEVS questions assumed a priori to cover religion exclusively (*10*), we used EFA to allow the patterns of variation to emerge from all the WEVS data. Previous studies also tend to cover a relatively short time span. In contrast, our use of unsupervised factor analysis, from five waves of all available countries in the WVS, allows comparison of a century of change in cultural values across multiple non-Western religions and cultures. Specifically, we were able to test whether GDP per capita in decade *t* predicts the secular values of people born in subsequent decades.

Using birth year data from the WEVS, we find that the average value of our extracted factors, such as secularization or tolerance of divorce, abortion, and homosexuality, has coherence that distinguishes one generation from another through time. That is, the persistence of generational values is consistent with both the theory that intergenerational change is a coherent mode of value change (*5*, *17*) and the theory that demographic shifts, rather than economics, drive modern cultural change (*1*).

Because religious beliefs and practices are culturally inherited (*30*), there may be positive feedback in secularization among generations raised with reduced exposure to religious practices (*28*, *29*). These generational patterns affirm that cultural values change at the population scale; this is consistent with, for example, WEVS evidence for acculturation of migrants on a time scale on the order of a decade (*31*, *32*).

Controlling for shared history did not substantially alter our findings, which accords with the observed correlations being either negligible or already very old by the start of the 20th century. The correlation between economic development and secularization, robust by the end of the 20th century, did not yet exist at the beginning of the 20th century (Fig. 3). In contrast, given the persistence of traditional values (*7*, *33*) and specifically the deep ancestry of religious prohibition and cooperation (*4*, *34*), the relationship between secularization and tolerance could be ancient, and we observe this already by the early 20th century.

The pace of change and its causality are important dimensions for future study. Studies of deep “cultural ancestry” on a time scale of centuries or millennia (*30*, *35*) have suggested, for instance, that ancient religious practices preceded the subsequent development of socioeconomic stratification (*36*). In the 21st century, however, cultural transmission has been accelerated and reconfigured by technological changes (*33*), and future tipping points may not be readily predicted from 20th century trends (*1*). Acceptance of gay marriage in Western countries, for example, reached 85% by 2017 among religiously unaffiliated Americans (*37*). In sub-Saharan Africa, people describing religion as their only belief system declined gradually from 75 to 13% over the 20th century (*38*), and certain regions have seen recent abrupt declines in female genital modification (*35*). These unanticipated changes remind us to be open to unprecedented causal pathways between development, religion, and tolerance in the future.

## MATERIALS AND METHODS

We used the WEVS data in three steps. First, we used EFA to automatically extract cultural “factors” from the last five waves of the WEVS data, collected between 1990 and 2014, yielding nine major factors as linear combinations of survey question responses. Rather than defining secularization in terms of a narrow variable such as low church attendance (*9*, *13*, *29*), or constrained to Western-Christian countries (*13*, *28*), we used a multi-item composite variable (*10*) to capture secularization as reduced importance of religion in people’s values across a diversity of cultures and religions in the sample of 109 nations represented in the sample.

Second, we used birth years of survey respondents to extract estimates of value change over the entire 20th century. On the basis of observations that an individual’s formative years are a good predictor of relative lifetime values (*5*, *39*), we subdivided the WEVS data by decade of birth to estimate values in decades predating the WEVS surveys. Because formative years may vary, we systematically tested three regressions: one assuming that the formative years are in the first decade of life (childhood), another assuming the second decade (teenage years), and another assuming the third (young adult). In all cases, our tests confirmed that decade of birth had a marked influence on our measure of secularization, and we studied time-seriated data across 109 countries and the 10 decades of the 20th century.

Third, we looked at how the 10-decade time series of secularization in each country relates to the record of change in GDP during the corresponding period. With respect to the well-known difficulties in establishing causation with observational data (*9*, *10*, *28*), here, we only say that changes in certain variables precede others, which can nevertheless rule out specific models of causation in favor of others.

### Cultural values surveys

The WVS (worldvaluessurvey.org) was administered to cumulatively 329,723 participants in six waves, in all nations accessible at the time of each wave, through a questionnaire administered through individual, face-to-face interviews in local languages. The WVS contains about 150 questions relating to cultural values, plus additional questions to collect demographic information. The WVS was carried out in almost 100 countries. At least 1000 people from each country were surveyed using a stratified sampling method to ensure a fair demographic representation (*7*).

The WVS was carried out in five waves since 1990, with one administered every 5 years. In early WVS waves, the populations of India, China, Nigeria, rural areas, and illiterate population were undersampled. Out of the 150 cultural value questions, 64 are common to all five waves since 1990 (table S3) and make up the core questions that are the focus of our analysis. As the questions varied in form (some binary, some Likert scale), we then recoded the WVS data by normalizing all scores to mean of zero across the whole WVS and set the variance to unity so that the variances are comparable. Missing data were limited, so missing values could be mean imputed without introducing bias.

The EVS contains the same core 64 questions found in the WVS, so the combined data set WEVS comprises these questions. The EVS covers 48 European countries (one EVS wave also included the United States). This increases the total number of unique countries to 109 (table S2).

### Exploratory factor analysis

We identified nine distinct cultural factors in the WEVS data using EFA, which assumes that each observed variable in the data set is a weighted linear combination of hidden factors (*40*)

$$y_{n}=w_{n,1}F_{1}+w_{n,2}F_{2}+\ldots +w_{n,m}F_{m}+\epsilon_{n}$$ (3)

where *y*_*n*_ is observed variable *n*, *F*_*m*_ is hidden factor *m*, *w*_*n*,*m*_ is the contribution of factor *F*_*m*_ to variable *y*_*n*_, and *ϵ*_*n*_ is the residual for variable *n*. This model was fit to the data using maximum likelihood. Nine factors were chosen based on the “Very Simple Structure” criterion (*41*), which maximizes the simple structure of the factor loading matrix for ease of interpretation.

Of these nine factors (see tables S4 to S12 for loadings), we focus on two, which we designated for each country *i* as secularization (*S*_*t*_) and tolerance of behavioral norms such as homosexuality and abortion (*V*_*t*_). The secularization factor was the one that explained the most variance in the EFA, and this factor was highly loaded upon WVS questions including “How important in your life is religion?,” “How important is God in your life?,” “Are you a religious person?,” “How often do you attend religious services,?” “How much confidence do you have in the Church?,” and “Is religious faith an important quality to instill in a child?” Tolerance, *V*_*t*_, was highly loaded upon questions concerning the respondents’ attitude toward homosexuality, divorce, suicide, and abortion.

### Economic development (historic GDP data)

We used historical data on GDP per capita (in 1990 US$) for the 20th century (1900–2000) provided by the Maddison Project (*19*). Because our WEVS analysis was resolved by decade, we correspondingly averaged the observed GDP per capita by decade from 1900 to 2010. Only six countries in the WEVS were not present in the Maddison data (Northern Ireland, Malta, Luxembourg, Iceland, Andorra, and Cyprus), yielding 103 countries with 11-point time series for GDP per capita. Historical GDP data are missing for certain countries, such as sub-Saharan Africa (for example, Nigeria and Burkina Faso) and former Soviet states (for example, Ukraine, Belarus, and Russia). For historical continuity, the following countries were considered the same: Cape Colony has been equated with South Africa, Holland with the Netherlands, Eritrea with Ethiopia, north and central Italy with Italy, and Great Britain and England with the United Kingdom.

### Tertiary education enrollment

We used tertiary education enrollment rates as a proxy measurement for science education. The “Barro-Lee Educational Attainment” data set gives time series for tertiary enrollment, taken mainly from census data and from intergovernmental organizations, and stretches back to 1820 in the most recent edition (*21*). We took the average rate of enrollment in each decade to correspond to the cultural values data, which is in decadal increments. The coverage is less comprehensive than the WEVS, with only 74 countries covered. Data for most non-Russian former Soviet states are missing because most were not independent states for most of the 20th century; the same is true for Yugoslavia. Some small countries or semiautonomous regions of another country are also missing, such as Northern Ireland. Finally, poorer countries—mainly Islamic or African ones—are missing because tertiary educational enrollment statistics could not be obtained.

### Language categories (proxy for cultural relatedness)

To avoid Galton’s problem, we have to control for shared culture. Often, this is done using language phylogenies (*22*), but this requires all societies under study to be from the same language tree with the requisite branch lengths calculated (*42*). The countries in our global sample speak languages from many different language families, which rules out the use of phylogenetic trees. To control for cultural history, *h*, in the time-lagged regressions, we discretely categorized the countries based on language families and treat it as a random effect. These data were taken from the Ethnologue database (*43*), which documents all known extant languages, and the countries in which they are currently the predominant language. The 109 countries were categorized into the following language families (number of countries): Albanian (2), Semitic (17), Italic (23), Greek-Armenian (3), Germanic (23), Turkic (6), Indo-Aryan (4), Balto-Slavic (14), Sino-Tibetan (3), Uralic (3), Kartvelian (1), Austronesian (3), Japonic (1), Niger-Congo (3), Korean (1), Tai (1), and Austroasiatic (1). Table S17 contains the language group assigned to each country.

### Use of birth cohort to extend data set through time

The WVS component of the combined WEVS data set was carried out during five distinct “waves,” carried out at approximately 5-year intervals, between 1990 and 2015. This provides a maximum of five data points per country (not all countries participated in all five waves) in a time series reaching back only 25 years. Given the recorded decade of birth of the survey respondents, however, we can, by assumptions confirmed below, extend these data back to represent all decades of the 20th century. This yields a matrix *S*_*t*,*p*_ of values for each country, with decade of birth, *t*, and survey period, *p*, as the rows and columns, respectively (for inclusion, a birth cohort must contain at least 100 individuals). To account for birth cohorts that are not represented in all time periods, which could otherwise bias the mean across time periods, we imputed the missing values using the following linear model

$$S_{t,p}=\mu_{p}+\alpha_{p}t$$ (4)

where *t* is the birth decade, *p* is the survey period, and μ_*p*_ and α are the estimated slope and intercept, respectively, for imputation of the missing value(s). Once missing values were imputed, we then defined *S*_*t*_ for each birth decade, *t*, as the mean across all survey periods, *p*. The result is a 10-point time series over the past century (rather than 5 points over 25 years) for the 109 countries in the WEVS, with some countries having only partially complete time series (for example, Nigeria has data from only seven decades). Importantly though, these values should not be interpreted as the true values, which would have been measured had the WEVS existed in earlier decades of the 20th century, except possibly when no period effect is present.

We tested the preservation of generational trends in cultural values (*5*) by using a likelihood ratio test to determine whether an interaction term between birth decade and survey period provides explanatory value for the data. Specifically

$$H_{0}:S_{t,p}=\alpha_{0}+\alpha_{1}t+\sum_{2}^{i=P}\alpha_{i}P_{i}+\epsilon$$ (5)

$$H_{1}:S_{t,p}=\beta_{0}+\beta_{1}t+\sum_{2}^{i=P}\beta_{i}P_{i}+\sum_{2}^{j=P}\beta_{j+P}P_{j}t+\epsilon$$ (6)

where *S*_*t*,*p*_ is secularization, but could also be tolerance of homosexuality and abortion *V*_*t*,*p*_. Each country was subjected to this test. We reported the likelihood ratio and the proportion of the variance explained by *H*_1_, not explained by *H*_0_. Further, using the χ^2^ distribution, we calculated asymptotic significance values to quantify the evidence that *H*_1_ was a better explanation for the WEVS data than *H*_0_, that is, whether estimates for each birth decade *t* were independent of survey period *p*. This test was carried out for 79 countries because we were limited to those who appeared in two or more waves of the WEVS.

### Multilevel time-lagged linear regressions

We chose a time-lagged model to express secularization (*S*_*t*_) as a function of historical development (GDP_*t*−*y*_) while controlling for historical secularization data (*S*_*t*−*y*_), where *y* is the lag in decades. Unlike a standard time-lag test, however, which normally requires two long individual time series, we have many time series (103 countries) that have 10 points or fewer (limited to number of decades in the 20th century). To control for cultural non-independence between countries, which is a nested random effect, we categorized countries by language family—as the best available proxy for cultural similarity—designated by variable *h*_*i*_ for country *i*. This amounted to two nested random effects for each designated cultural heritage *h*, within each country *i*. To avoid multiple testing and low statistical power, we formulated a multilevel model to incorporate data from all countries into a single test

$$S_{t}\sim S_{t-y}+{\text{GDP}}_{t-y}+(1|h_{i})+\epsilon$$ (7)

where (1|*h*_*i*_) is the nested random effect for a country *i* from language category *h*, ϵ is the error, and we let *y* = 1, 2, or 3 decades. Using the control variable, *h*_*i*_, present in all of the time-lagged equations (Eqs. 1, 2, 7, and 12), we found that this nested random effect did not substantially change our results (Table 1 and tables S14 to S17). This indicated that religious change predicted economic change while controlling for language as a proxy for shared history.

To deal with missing data in the GDP_*t*_ and/or *S*_*t*_ time series for certain countries, we chose to omit the missing values rather than attempt to impute them without an obvious universal model to describe how secularization or GDP changes. However, despite omitting variables, we still obtained sizable contributions from the major cultural groups (except for the ex-Soviet states that lack credible GDP data before 1990). We also reported the number of countries represented in the data and the number of total data points; both depended on the time lag used.

To test the alternative hypothesis that economic development precedes secularization, we ran a similar test to see whether *S*_*t*_ in a birth cohort predicted GDP *y* decades later

$${\text{GDP}}_{t}\sim {\text{GDP}}_{t-y}+S_{t-y}+(1|h_{i})+\epsilon$$ (8)

We also tested the effect of tolerance of behaviors such as homosexuality and abortion, *V*, on either *S* or GDP. We added *V* as a control in the time-lagged regressions

$$S_{t}\sim S_{t-y}+{\text{GDP}}_{t-y}+V_{t-y}+(1|h_{i})+\epsilon$$ (9)

$${\text{GDP}}_{t}\sim {\text{GDP}}_{t-y}+S_{t-y}+V_{t-y}+(1|h_{i})+\epsilon$$ (10)

We also wanted to test the effect of advanced education *E*, so we similarly added a variable representing the tertiary education enrollment rate. Once again, testing a lag of *y* = 1, 2, or 3 decades

$$S_{t}\sim S_{t-y}+{\text{GDP}}_{t-y}+E_{t-y}+(1|h_{i})+\epsilon$$ (11)

$${\text{GDP}}_{t}\sim {\text{GDP}}_{t-y}+S_{t-y}+E_{t-y}+(1|h_{i})+\epsilon$$ (12)

We normalized *S*_*t*_ and *V*_*t*_ so that the SD of each is equal to 1. This allowed us to state the dollar improvement in GDP resulting from 1 SD change in both *S*_*t*_ and *V*_*t*_.

### Robustness checks

When comparing GDP data versus our estimates of secularization (*S*_*t*_) for given birth decade, *t*, we make no assumption about the age at which a birth cohort begins to affect the economy; economic development can affect cultural values during formative years, whereas people will not normally influence the economy until they are older. To ensure that our results are robust to this uncertainty, we ran the S-GDP regressions considering coincidence points between development and secularization in birth cohorts: childhood (+0), teenage years (+10), and twenties (+20). The results in table S15 show that, under all of these scenarios, secularization precedes economic development and not the other way around.

We also tested the robustness of our multilevel, time-lagged regressions to ensure that random noise in the EFA factors did not affect the regression results. To do this, instead of defining secularization with EFA factors, we defined secularization as the simple mean of six relevant WEVS variables (see table S16), each normalized to mean zero and unit variance. We found that the predictive structure that emerges (table S16) is the same as when we used the factors derived through EFA.

## Supplementary Material

http://advances.sciencemag.org/cgi/content/full/4/7/eaar8680/DC1

## SUPPLEMENTARY MATERIALS

Supplementary material for this article is available at http://advances.sciencemag.org/cgi/content/full/4/7/eaar8680/DC1 Section S1. WVS and EVS Section S2. Exploratory factor analysis Section S3. Cultural factor loadings Table S1. Participation in the different waves of the WVS and EVS. Table S2. Participating countries in the WEVS. Table S3. Questions common to all eight waves of the WVS and EVS. Table S4. Secularization. Table S5. Institutional confidence. Table S6. Openness to intrinsic differences. Table S7. Prosociality. Table S8. Interest of politics. Table S9. Wellbeing. Table S10. Political engagement. Table S11. Tolerance of prohibited behaviors. Table S12. Openness to extrinsic differences. Table S13. Independence of birth decade, *t*, versus WEVS phase, *p*, for secularization, *S*_*t*,*p*_, and tolerance, *V*_*t*,*p*_. Table S14. Multilevel time-lagged linear models (see Materials and Methods) demonstrating that secularization predicts GDP and not vice versa (models 1 to 6); tolerance predicts GDP better than secularization (models 7 to 12) and education predicts future GDP, but not secularization (models 13 to 18). Table S15. Time-lagged models, models 1 to 6 (see Materials and Methods), of *S* versus GDP for cohorts in their first decade or childhood (*y* = 0 decades, top row), teenage years (*y* = 1 decade, middle row), and twenties (*y* = 2 decades, bottom row). Table S16. Multilevel time-lagged models, but with secularization (*S*_*alt*_) measured using the average of six indicators, which are subjectively associated with religiosity. Table S17. Language categories assigned to WEVS countries, using Ethnologue data. Fig. S1. The ordered factor loadings on WEVS survey questions, following EFA analysis with oblique rotation.
