# Supplementary material for: Religious change preceded economic change in the 20th century
Source: Sci Adv. 2018 Jul 18;4(7):eaar8680. doi: 10.1126/sciadv.aar8680 (PMC6051740; doi:10.1126/sciadv.aar8680)
Supplement: http://advances.sciencemag.org/cgi/content/full/4/7/eaar8680/DC1 [file supp_4_7_eaar8680__index.html]

Science Advances | Science Advances

## Supplementary Materials

**This PDF file includes:**

- Section S1. WVS and EVS
- Section S2. Exploratory factor analysis
- Section S3. Cultural factor loadings
- Table S1. Participation in the different waves of the WVS and EVS.
- Table S2. Participating countries in the WEVS.
- Table S3. Questions common to all eight waves of the WVS and EVS.
- Table S4. Secularization.
- Table S5. Institutional confidence.
- Table S6. Openness to intrinsic differences.
- Table S7. Prosociality.
- Table S8. Interest of politics.
- Table S9. Wellbeing.
- Table S10. Political engagement.
- Table S11. Tolerance of prohibited behaviors.
- Table S12. Openness to extrinsic differences.
- Table S13. Independence of birth decade, *t*, versus WEVS phase, *p*, for secularization, *S**t*,*p*, and tolerance, *V**t*,*p*.
- Table S14. Multilevel time-lagged linear models (see Materials and Methods) demonstrating that secularization predicts GDP and not vice versa (models 1 to 6); tolerance predicts GDP better than secularization (models 7 to 12) and education predicts future GDP, but not secularization (models 13 to 18).
- Table S15. Time-lagged models, models 1 to 6 (see Materials and Methods), of *S* versus GDP for cohorts in their first decade or childhood (*y* = 0 decades, top row), teenage years (*y* = 1 decade, middle row), and twenties (*y* = 2 decades, bottom row).
- Table S16. Multilevel time-lagged models, but with secularization (*S**alt*) measured using the average of six indicators, which are subjectively associated with religiosity.
- Table S17. Language categories assigned to WEVS countries, using Ethnologue data.
- Fig. S1. The ordered factor loadings on WEVS survey questions, following EFA analysis with oblique rotation.

Download PDF

**Files in this Data Supplement:**

- Adobe PDF - aar8680\_SM.pdf
