# Supplementary material for: Religious change preceded economic change in the 20th century
Source: Sci Adv. 2018 Jul 18;4(7):eaar8680. doi: 10.1126/sciadv.aar8680 (PMC6051740; doi:10.1126/sciadv.aar8680)
Supplement: http://advances.sciencemag.org/cgi/content/full/4/7/eaar8680/DC1 [file aar8680_SM.pdf]

Science Advances
AAAS

advances.sciencemag.org/cgi/content/full/4/7/eaar8680/DC1

Supplementary Materials for

Religious change preceded economic change in the 20th century

Damian J. Ruck, R. Alexander Bentley*, Daniel J. Lawson

*Corresponding author. Email: rabentley@utk.edu

Published 18 July 2018, *Sci. Adv.* **4**, eaar8680 (2018)
DOI: 10.1126/sciadv.aar8680

This PDF file includes:

Section S1. WVS and EVS
Section S2. Exploratory factor analysis
Section S3. Cultural factor loadings
Table S1. Participation in the different waves of the WVS and EVS.
Table S2. Participating countries in the WEVS.
Table S3. Questions common to all eight waves of the WVS and EVS.
Table S4. Secularization.
Table S5. Institutional confidence.
Table S6. Openness to intrinsic differences.
Table S7. Prosociality.
Table S8. Interest of politics.
Table S9. Wellbeing.
Table S10. Political engagement.
Table S11. Tolerance of prohibited behaviors.
Table S12. Openness to extrinsic differences.
Table S13. Independence of birth decade, t, versus WEVS phase, p, for secularization, $S_{t,p}$, and tolerance, $V_{t,p}$.
Table S14. Multilevel time-lagged linear models (see Materials and Methods) demonstrating that secularization predicts GDP and not vice versa (models 1 to 6); tolerance predicts GDP better than secularization (models 7 to 12) and education predicts future GDP, but not secularization (models 13 to 18).
Table S15. Time-lagged models, models 1 to 6 (see Materials and Methods), of S versus GDP for cohorts in their first decade or childhood (y = 0 decades, top row), teenage years (y = 1 decade, middle row), and twenties (y = 2 decades, bottom row).
Table S16. Multilevel time-lagged models, but with secularization (S_{alt}) measured using the average of six indicators, which are subjectively associated with religiosity.
Table S17. Language categories assigned to WEVS countries, using Ethnologue data.
Fig. S1. The ordered factor loadings on WEVS survey questions, following EFA analysis with oblique rotation.

Section S1. WVS and EVS

The WVS and EVS were administered in waves. There have been five waves of the WVS and three of the EVS since 1990 (see table S1 for participation figures). A WVS and an EVS wave from the early eighties were excluded because the questions did not sufficiently overlap with those from later waves and they were only administered in a few, mainly Western, countries. The total number of participants since 1990 is 475,342.

In total the survey was carried out in 109 unique countries representing over 90% of the worlds population and all geographical regions (a list of the participating countries can be found in table S13). In order to set the data in a form amenable to the EFA regime, all categorical response data were removed, leaving just data of continuous and ordinal types (e.g. binary and Likert scales). All of the variables were transformed to zero mean and unit variance. Finally, missing values were mean imputed. We are able to aggregate the World Value Survey (WVS) and the European Value Survey (EVS) together —we refer to the combined data as WEVS —because the 64 core questions, which are common to the later five waves of the WVS, are present in the later three waves of the EVS. These questions are contained in table S3.

Table S1. Participation in the different waves of the WVS and EVS.

EVS/WVS	Wave	Years	No. Countries	No. Participants
WVS	2	1990-1994	18	24,558
EVS	2	1990-1994	29	38,213
WVS	3	1995-1998	56	76,075
EVS	3	1995-1999	33	41,125
WVS	4	1999-2004	41	60,045
EVS	4	2005-2010	47	66,281
WVS	5	2005-2009	58	83,975
WVS	6	2010-2014	60	85,070

Table S2. Participating countries in the WEVS.

Albania	Cyprus (T)	Iran	Morocco	South Africa
Algeria	Czech Rep.	Iraq	Netherlands	South Korea
Andorra	Denmark	Ireland	New Zealand	Spain
Argentina	Dominican Rep.	Israel	Nigeria	Sweden
Armenia	Ecuador	Italy	North Ireland	Switzerland
Australia	Egypt	Japan	Norway	Taiwan
Austria	El Salvador	Jordan	Pakistan	Tanzania
Azerbaijan	Estonia	Kazakhstan	Palestine	Thailand
Bahrain	Ethiopia	Kosovo	Peru	Trinidad and Tobago
Bangladesh	Finland	Kuwait	Philippines	Tunisia
Belarus	France	Kyrgyzstan	Poland	Turkey
Belgium	Georgia	Latvia	Portugal	Uganda
Bosnia	Germany	Lebanon	Puerto Rico	Ukraine
Brazil	Ghana	Libya	Qatar	United States
Bulgaria	Great Britain	Lithuania	Romania	Uruguay
Burkina Faso	Greece	Luxembourg	Russia	Uzbekistan
Canada	Guatemala	Macedonia	Rwanda	Venezuela
Chile	Hong Kong	Malaysia	Saudi Arabia	Viet Nam
China	Hungary	Mali	Serbia and Montenegro	Yemen
Colombia	Iceland	Malta	Singapore	Zambia
Croatia	India	Mexico	Slovakia	Zimbabwe
Cyprus (G)	Indonesia	Moldova	Slovenia	

Table S3. Questions common to all eight waves of the WVS and EVS.

Important in life: Family	Important in life: Friends
Important in life: Leisure time	Important in life: Politics
Important in life: Work	Important in life: Religion
Feeling of happiness	State of health (subjective)
Important child qualities: independence	Important child qualities: hard work
Important child qualities: feeling of responsibility	Important child qualities: imagination
Important child qualities: tolerance and respect for other people	Important child qualities: thrift saving money and things
Important child qualities: determination perseverance	Important child qualities: religious faith
Important child qualities: unselfishness	Important child qualities: obedience
Neighbours: People of a different race	Neighbours: Heavy drinkers
Neighbours: Immigrants/foreign workers	Neighbours: People who have AIDS
Neighbours: Drug addicts	Neighbours: Homosexuals
Most people can be trusted	Satisfaction with your life
How much freedom of choice and control	Jobs scarce: Men should have more right to a job than women
Jobs scarce: Employers should give priority to (nation) people than immigrants	Being a housewife just as fulfilling
Future changes: Greater respect for authority	Interest in politics
Political action: signing a petition	Political action: joining in boycotts
Political action: attending lawful/peaceful demonstrations	Self positioning in political scale
Income equality	Private vs state ownership of business
Government responsibility	Competition good or harmful
Confidence: Churches	Confidence: Armed Forces
Confidence: The Press	Confidence: Labour Unions
Confidence: The Police	Confidence: Parliament
Confidence: The Civil Services	Confidence: The Government
Confidence: The Political Parties	Confidence: Major Companies
How often do you attend religious services	Religious person
How important is God in your life	Justifiable: claiming government benefits
Justifiable: avoiding a fare on public transport	Justifiable: cheating on taxes
Justifiable: someone accepting a bribe	Justifiable: homosexuality
Justifiable: prostitution	Justifiable: abortion
Justifiable: divorce	Justifiable: euthanasia
Justifiable: suicide	How proud of nationality

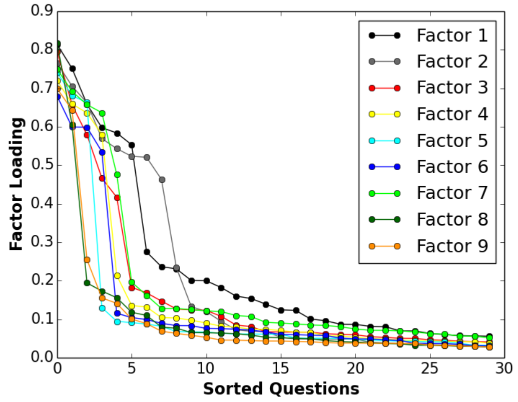

Fig. S1. The ordered factor loadings on WEVS survey questions, following EFA analysis with oblique rotation.

Section S2. Exploratory Factor Analysis (EFA)

Exploratory Factor Analysis (EFA) is an unsupervised method to uncover any underlying structure in large multivariate dataset. EFA assumes each observed variable in the dataset is a weighted linear combination of some set of hidden factors that are to be predicted. This procedure is put more formally in Equation S1; where y_n is variable n, F_m is hidden factor m, $w_{n,m}$ is the contribution of factor F_m to variable y_n and ϵ_n is the error term for variable n. We fit this model using maximum likelihood (41):

$$y_n = w_{n,1}F_1 + w_{n,2}F_2 + ... + w_{n,m}F_m + \epsilon_n \tag{S1}$$

The second part of the EFA regime is an oblique rotation. Rotation acts to improve the interpretability of factors by approaching a simple structure in the factor loading matrix. A factor loading matrix has a perfect simple structure if the following criteria are met (42)

1. Each factor has a small subset of variables with a large factor loadings.
2. The remaining factor loadings should be vanishingly small.
3. Each variable should have a large factor loading on one factor only.

The approximate simple structure, obtained using the WEVS data, is illustrated in Figure 1 where each factor is highly loaded by a small set of variables with a bulk having small loadings. We use an "oblimin" rotation, an oblique rotation, rather than an orthogonal rotation. An oblique rotation relaxes the orthogonality constraint implicit in EFA, so it allows greater freedom when obtaining simple structure. Plus, the orthogonal assumption does not correspond with the real world, where definable cultural values are often correlated.

Table S4. **Secularization** - Factor 1.

WVS question	loading
Important in life: Religion	0.82
How important is God in your life	-0.82
Religious person	0.71
How often do you attend religious services	0.65
Confidence: The Churches	0.64
Important child qualities: Religious faith	0.53

Given our focus on the interpretability of factors by maximizing simple structure in the factor loading matrix, we chose the number of factors to retain accordingly. The "Very Simple Structure" (VSS) algorithm selects the number of factors which maximizes simple structure (42), defined as

$$\Sigma_{res} = \Sigma - SS^T \quad \text{(S2)}$$

$$VSS = 1 - \frac{\Sigma_{res}}{\Sigma} \quad \text{(S3)}$$

where Σ is the real correlation matrix of the WEVS data and S is the simplified factor loading matrix. The VSS score can take values between one and zero; it takes a value of one when simple structure is present and zero when not. VSS is defined for a given "complexity," c, which is the number of factor loadings retained when defining S; for example, if $c = 5$, then all but the highest five factor loadings are set to zero. Σ_{res} is the error created when the simplified correlation matrix SS^T is compared to the real one (Σ). If σ can be recreated using just the c highest factor loadings, then this is the definition of a simple structure and VSS will be close to one.

All aspects of the EFA regime were implemented in R using the 'psych' package (http://personality-project.org/r/psych/).

Section S3. Cultural factor loadings

Tables S4-S12 below list the most significant sets of WEVS questions, and their factor loadings, for Factors 1-9 respectively.

Table S5. **Institutional confidence**.- Factor 2.

WVS question	loading
Confidence: Parliament	0.81
Confidence: The Government	0.73
Confidence: The Civil service	0.70
Confidence: Political Parties	0.70
Confidence: The police	0.58
Confidence: Labour Unions	0.53
Confidence: Major Companies	0.51
Confidence: The press	0.50
Confidence: The armed forces	0.44

Table S6. **Openness to intrinsic differences**.- Factor 3.

WVS question	loading
Neighbors: Drug addicts	0.68
Neighbors: Homosexuals	0.63
Neighbors: Heavy drinkers	0.58
Neighbors: People who have AIDS	0.55

Table S7. **Prosociality**.- Factor 4.

WVS question	loading
Justifiable: Cheating on taxes	0.72
Justifiable: Avoiding a fare on public transport	0.66
Justifiable: Someone accepting a bribe	0.64
Justifiable: Claiming government benefits (not entitled)	0.58

Table S8. **Interest of politics**.- Factor 5.

WVS question	loading
Important in life: Politics	0.88
Interest in politics	0.59

Table S9. **Wellbeing**.- Factor 6.

WVS question	loading
Satisfaction with your life	-0.71
Feeling of happiness	0.67
State of health (subjective)	0.50
Freedom of choice and control over own life	-0.46

Table S10. **Political engagement**.- Factor 7

WVS question	loading
Joining in boycotts	0.74
Attending peaceful demonstrations	0.71
Signing a petition	0.66

Table S11. **Tolerance of prohibited behaviors**.- Factor 8.

WVS question	loading
Justifiable: Divorce	0.74
Justifiable: Abortion	0.73
Justifiable: Homosexuality	0.68
Justifiable: Prostitution	0.62
Justifiable: Euthanasia	0.55
Justifiable: Suicide	0.52

Table S12. **Openness to extrinsic differences**.- Factor 9.

WVS question	loading
Neighbors: People of a different race	0.72
Neighbors: Immigrants/foreign workers	0.66

Table S13. Independence of birth decade, t, versus WEVS phase, p, for secularization, $S_{t,p}$, and tolerance, $V_{t,p}$. Entries show proportion of variance explained by H_1. Likelihood ratio, H_0 vs. H_1, in parentheses (Materials and Methods). Bonferroni corrected significance of 5%: ** $p < 0.0006$.

Country	$S_{t,p}$	$V_{t,p}$	Country	$S_{t,p}$	$V_{t,p}$
Albania	0.233(1.557)	0.006(1.024)	Latvia	0.133(1.423)**	0.007(1.054)
Algeria	0.038(1.076)	0.000(1.004)	Lithuania	0.021(1.102)	0.004(1.026)
Argentina	0.013(1.069)	0.021(1.127)	Macedonia	0.061(1.219)	0.116(1.275)
Armenia	0.002(1.019)	0.020(1.317)**	Malaysia	0.113(1.047)	0.062(1.402)
Australia	0.005(1.024)	0.006(1.036)	Mexico	0.014(1.060)	0.010(1.054)
Austria	0.004(1.033)	0.024(1.200)	Moldova	0.053(1.144)	0.042(1.165)
Azerbaijan	0.144(1.190)	0.000(1.002)	Morocco	0.052(1.120)	0.109(1.282)**
Bangladesh	0.013(1.005)	0.307(1.067)	Netherlands	0.022(1.120)	0.052(1.163)
Belarus	0.025(1.107)	0.024(1.151)	New Zealand	0.034(1.056)	0.054(1.160)
Belgium	0.038(1.223)	0.044(1.217)	Nigeria	0.148(1.143)	0.113(1.326)**
Bosnia	0.365(1.922)**	0.011(1.067)	Norway	0.017(1.119)	0.008(1.108)
Brazil	0.097(1.163)	0.001(1.005)	Pakistan	0.048(1.058)	0.032(1.102)
Bulgaria	0.039(1.207)	0.026(1.186)	Peru	0.039(1.145)	0.049(1.202)
Canada	0.014(1.105)	0.010(1.035)	Philippines	0.022(1.009)	0.044(1.077)
Chile	0.036(1.204)	0.049(1.707)**	Poland	0.005(1.024)	0.023(1.120)
China	0.008(1.106)	0.063(1.316)**	Portugal	0.022(1.175)	0.010(1.091)
Colombia	0.004(1.008)	0.001(1.004)	Puerto Rico	0.000(1.001)	0.002(1.012)
Croatia	0.114(1.127)	0.006(1.036)	Romania	0.081(1.449)**	0.058(1.365)**
Czech Rep.	0.039(1.186)	0.018(1.107)	Russia	0.070(1.226)	0.008(1.063)
Denmark	0.096(1.495)**	0.016(1.105)	Rwanda	0.030(1.234)	0.001(1.000)
Egypt	0.015(1.115)	0.010(1.091)	Serbia	0.074(1.371)**	0.038(1.276)**
Estonia	0.070(1.163)	0.001(1.005)	Singapore	0.006(1.048)	0.003(1.026)
Finland	0.021(1.107)	0.049(1.407)	Slovakia	0.024(1.122)	0.002(1.017)
France	0.039(1.139)	0.005(1.023)	Slovenia	0.008(1.028)	0.016(1.178)
Georgia	0.007(1.101)	0.037(1.192)	South Africa	0.058(1.122)	0.003(1.033)
Germany	0.072(1.500)**	0.063(1.639)**	South Korea	0.061(1.266)	0.016(1.189)
Ghana	0.363(1.102)	0.094(1.056)	Spain	0.002(1.033)	0.008(1.117)
Great Britain	0.029(1.212)	0.005(1.024)	Sweden	0.036(1.119)	0.033(1.275)
Greece	9.271(1.000)	0.018(1.196)	Switzerland	0.018(1.142)	0.020(1.187)
Hong Kong	0.230(1.050)	0.003(1.016)	Taiwan	0.006(1.034)	0.004(1.033)
Hungary	0.066(1.547)	0.007(1.021)	Thailand	0.041(1.205)	0.162(1.253)
India	0.151(1.413)**	0.008(1.101)	Trinidad	1.889(1.000)	0.022(1.050)
Indonesia	0.043(1.104)	0.002(1.004)	Turkey	0.064(1.136)	0.092(1.234)
Iran	0.005(1.016)	0.040(1.203)	Ukraine	0.120(1.254)	0.018(1.120)
Iraq	0.088(1.086)	0.023(1.213)	United States	0.013(1.063)	0.006(1.026)
Ireland	0.015(1.203)	0.039(1.393)	Uruguay	0.008(1.055)	0.109(1.379)
Italy	0.004(1.014)	0.005(1.031)	Venezuela	0.146(1.149)	0.024(1.047)
Japan	0.026(1.123)	0.026(1.154)	Viet Nam	0.078(1.013)	0.828(1.079)
Jordan	0.035(1.103)	0.005(1.027)	Zimbabwe	0.321(1.031)	0.000(1.003)
Kyrgyzstan	0.003(1.002)	0.311(1.126)			

Table S14. Multilevel time-lagged linear models (see Materials and Methods) demonstrating that secularization predicts GDP and not vice versa (models 1 to 6); tolerance predicts GDP better than secularization (models 7 to12) and education predicts future GDP, but not secularization (models 13 to 18). Row labels are abbreviated as follows: "dep" is the dependent variable, GDP is historical GDP, S is effect of past secularization, E is historical education, V is historical tolerance, y is time lag in decades, N is the number of data points, n is the number of unique countries, i is variance explained by the country random effect, h is variance explained by cultural-history and R^2 is total variance explained. Standard errors are in parentheses. Bonferroni corrected significance: * $p < 0.1$; ** $p < 0.05$; *** $p < 0.01$.

	M1	M2	M3	M4	M5	M6
dep	S	S	S	GDP	GDP	GDP
GDP	0 (0.01)	-0.02 (0.03)	0.08 (0.06)	0.98 (0.02)***	0.87 (0.04)***	0.85 (0.05)***
S	1.02 (0.01)***	0.97 (0.02)***	0.75 (0.02)***	0.1 (0.01)***	0.28 (0.03)***	0.5 (0.03)***
y	1	2	3	1	2	3
N	417	324	229	497	469	387
n	101	95	83	96	101	101
i	0.05***	0.14***	0.34***	0.08***	0.21***	0.37***
h	0.05**	0.14***	0.3***	0.02	0.12*	0.19
R^2	0.99	0.99	0.99	0.92	0.89	0.95

	M7	M8	M9	M10	M11	M12
dep	S	S	S	GDP	GDP	GDP
GDP	-0.01 (0.02)	-0.01 (0.03)	0.08 (0.06)	0.95 (0.02)***	0.78 (0.04)***	0.71 (0.05)***
S	1.01 (0.02)***	0.99 (0.04)***	0.81 (0.06)***	0.03 (0.02)	0.01 (0.04)	0.07 (0.06)
V	0.01 (0.02)	-0.02 (0.03)	-0.04 (0.05)	0.09 (0.02)***	0.32 (0.04)***	0.44 (0.05)***
y	1	2	3	1	2	3
N	417	324	229	497	469	387
n	101	95	83	96	101	101
i	0.05***	0.14***	0.33***	0.07***	0.19***	0.31***
h	0.04	0.14***	0.28***	0.02	0.1	0.11
R^2	0.99	0.99	0.99	0.92	0.9	0.95

	M13	M14	M15	M16	M17	M18
dep	S	S	S	GDP	GDP	GDP
GDP	0 (0.02)	-0.04 (0.05)	0.1 (0.08)	0.98 (0.03)***	0.83 (0.05)***	0.75 (0.08)***
S	1.03 (0.01)***	0.97 (0.02)***	0.74 (0.03)***	0.07 (0.01)***	0.22 (0.02)***	0.4 (0.03)***
E	-0.06 (0.09)	0.14 (0.2)	0.18 (0.3)	0.39 (0.09)***	0.97 (0.19)***	1.31 (0.3)***
y	1	2	3	1	2	3
N	343	274	205	409	382	318
n	70	69	66	70	70	70
i	0.05***	0.15***	0.36***	0.05	0.16***	0.27***
h	0.05	0.12*	0.32**	0.03	0.09	0.16
R^2	0.99	0.99	0.99	0.94	0.91	0.94

Table S15. Time-lagged models, models 1 to 6 (see Materials and Methods), of S versus GDP for cohorts in their first decade or childhood ($y = 0$ decades, top row), teenage years ($y = 1$ decade, middle row) and twenties ($y = 2$ decades, bottom row). In each row, "dep" is the dependent variable, GDP is historical GDP, S is effect of past secularization, y is time lag in decades, N is number of data points, n is number of unique countries, i is variance explained by the country random effect, h is variance explained by cultural-history and R^2 is total variance explained. Standard errors are in parentheses. Bonferroni corrected significance: * $p < 0.1$; ** $p < 0.05$; *** $p < 0.01$. Significance: ** $p < 0.01$; *** $p < 0.001$.

	M1	M2	M3	M4	M5	M6
dep	S	S	S	GDP	GDP	GDP
GDP	0 (0.01)	-0.02 (0.03)	0.08 (0.06)	0.98 (0.02)***	0.87 (0.04)***	0.85 (0.05)***
S	1.02 (0.01)***	0.97 (0.02)***	0.75 (0.02)***	0.1 (0.01)***	0.28 (0.03)***	0.5 (0.03)***
y	1	2	3	1	2	3
N	417	324	229	497	469	387
n	101	95	83	96	101	101
i	0.05***	0.14***	0.34***	0.08***	0.21***	0.37***
h	0.05**	0.14***	0.3***	0.02	0.12*	0.19
R^2	0.99	0.99	0.99	0.92	0.89	0.95

	M1(+1)	M2(+1)	M3(+1)	M4(+1)	M5(+1)	M6(+1)
dep	S	S	S	GDP	GDP	GDP
GDP	-0.01 (0.01)	-0.02 (0.03)	0.02 (0.04)	0.99 (0.02)***	0.82 (0.04)***	0.84 (0.05)***
S	1.02 (0.01)***	0.97 (0.02)***	0.77 (0.03)***	0.11 (0.01)***	0.32 (0.03)***	0.4 (0.04)***
y	1	2	3	1	2	3
N	449	353	261	493	411	323
n	101	100	93	101	101	101
i	0.06***	0.15***	0.32***	0.08***	0.24***	0.34***
h	0.05***	0.13***	0.32***	0.03	0.15***	0.16
R^2	0.99	0.99	0.99	0.93	0.92	0.94

	M1(+2)	M2(+2)	M3(+2)	M4(+2)	M5(+2)	M6(+2)
dep	S	S	S	GDP	GDP	GDP
GDP	-0.02 (0.01)	-0.05 (0.02)	-0.04 (0.03)	0.95 (0.02)***	0.84 (0.04)***	0.81 (0.06)***
S	1.02 (0.01)***	0.98 (0.02)***	0.81 (0.03)***	0.14 (0.02)***	0.24 (0.03)***	0.33 (0.04)***
y	1	2	3	1	2	3
N	475	374	279	424	336	248
n	101	100	98	101	101	94
i	0.06***	0.15***	0.3***	0.11***	0.24***	0.34***
h	0.05**	0.13***	0.28***	0.04	0.14***	0.18**
R^2	0.99	0.99	0.99	0.94	0.91	0.93

Table S16. Multilevel time-lagged models, but with secularization (S_{alt}) measured using the average of six indicators,which are subjectively associated with religiosity. The results are similar to those using the secularization S derived using EFA: Secularization predicts development and not vice-versa. In each row, "dep" is the dependent variable, GDP is historical GDP, S is effect of past secularization, y is time lag in decades, N is number of data points, n is number of unique countries, i is variance explained by the country random effect, h is variance explained by cultural-history and R^2 is total variance explained. Standard errors are in parentheses. Bonferroni corrected significance: * $p < 0.1$; ** $p < 0.05$; *** $p < 0.01$.

	M1	M2	M3	M4	M5	M6
dep	S_{alt}	S_{alt}	S_{alt}	GDP	GDP	GDP
GDP	0 (0.01)	-0.02 (0.03)	0.04 (0.05)	0.99 (0.02)***	0.9 (0.04)***	0.84 (0.05)***
S_{alt}	1.02 (0.01)***	0.97 (0.02)***	0.81 (0.02)***	0.1 (0.01)***	0.28 (0.02)***	0.51 (0.03)***
y	1	2	3	1	2	3
N	478	387	289	559	512	427
n	101	98	86	98	101	101
i	0.05***	0.14***	0.3***	0.08***	0.21***	0.37***
h	0.04**	0.11**	0.25***	0.02	0.13**	0.21*
R^2	0.99	0.98	0.98	0.92	0.89	0.94

Table S17. Language categories assigned to WEVS countries, using Ethnologue data.

**country**	***language***	**country**	***language***	**country**	***language***
Albania	*Albania*	Greece	*Greek-Armenian*	Peru	*Italic*
Algeria	*Semitic*	Guatemala	*Italic*	Philippines	*Austronesian*
Andorra	*Italic*	Hong Kong	*Sino-Tibetan*	Poland	*Balto-Slavic*
Argentina	*Italic*	Hungary	*Uralic*	Portugal	*Italic*
Armenia	*Greek-Armenian*	Iceland	*Germanic*	Puerto Rico	*Italic*
Australia	*Germanic*	India	*Indo-Aryan*	Qatar	*Semitic*
Austria	*Germanic*	Indonesia	*Austronesian*	Romania	*Italic*
Azerbaijan	*Turkic*	Iran	*Indo-Aryan*	Russia	*Balto-Slavic*
Bahrain	*Semitic*	Iraq	*Semitic*	Rwanda	*Niger_Congo*
Bangladesh	*Indo-Aryan*	Ireland	*Germanic*	Saudi Arabia	*Semitic*
Belarus	*Balto-Slavic*	Israel	*Semitic*	Serbia and Montenegro	*Balto-Slavic*
Belgium	*Germanic*	Italy	*Italic*	Singapore	*Germanic*
Bosnia	*Balto-Slavic*	Japan	*Japan*	Slovakia	*Balto-Slavic*
Brazil	*Italic*	Jordan	*Semitic*	Slovenia	*Balto-Slavic*
Bulgaria	*Balto-Slavic*	Kazakhstan	*Turkic*	South Africa	*Germanic*
Burkina Faso	*Italic*	Kosovo	*Albania*	South Korea	*Korea*
Canada	*Germanic*	Kuwait	*Semitic*	Spain	*Italic*
Chile	*Italic*	Kyrgyzstan	*Turkic*	Sweden	*Germanic*
China	*Sino-Tibetan*	Latvia	*Balto-Slavic*	Switzerland	*Germanic*
Colombia	*Italic*	Lebanon	*Semitic*	Taiwan	*Sino-Tibetan*
Croatia	*Balto-Slavic*	Libya	*Semitic*	Tanzania	*Niger_Congo*
Cyprus (G)	*Greek-Armenian*	Lithuania	*Balto-Slavic*	Thailand	*Tai*
Cyprus (T)	*Turkic*	Luxembourg	*Italic*	Trinidad and Tobago	*Germanic*
Czech Rep.	*Balto-Slavic*	Macedonia	*Balto-Slavic*	Tunisia	*Semitic*
Denmark	*Germanic*	Malaysia	*Austronesian*	Turkey	*Turkic*
Dominican Rep.	*Italic*	Mali	*Italic*	Uganda	*Niger_Congo*
Ecuador	*Italic*	Malta	*Semitic*	Ukraine	*Balto-Slavic*
Egypt	*Semitic*	Mexico	*Italic*	United States	*Germanic*
El Salvador	*Italic*	Moldova	*Italic*	Uruguay	*Italic*
Estonia	*Uralic*	Morocco	*Semitic*	Uzbekistan	*Turkic*
Ethiopia	*Semitic*	Netherlands	*Germanic*	Venezuela	*Italic*
Finland	*Uralic*	New Zealand	*Germanic*	Viet Nam	*Austroasiatic*
France	*Italic*	Nigeria	*Germanic*	Yemen	*Semitic*
Georgia	*Kartvelian*	North Ireland	*Germanic*	Zambia	*Germanic*
Germany	*Germanic*	Norway	*Germanic*	Zimbabwe	*Germanic*
Ghana	*Germanic*	Pakistan	*Indo-Aryan*		
Great Britain	*Germanic*	Palestine	*Semitic*		
